# Regarding the new vascular reactivity index to norepinephrine

**DOI:** 10.1186/s13613-025-01519-y

**Published:** 2025-07-25

**Authors:** Enrique Monares-Zepeda, Christopher Barrera-Hoffmann

**Affiliations:** 1 Critical Care Medicine, Coordination of Intensive Therapy and Hemodynamics in Secondary-Level Hospitals, IMSS Bienestar, México City, México; 2 Critical Care Medicine, Hospital General de Zona, No. 71 IMSS, Veracruz México City, México

**Keywords:** Norepinephrine, Diastolic arterial pressure, Vascular responsiveness

Regarding the new vascular reactivity index to norepinephrine.

We would like to congratulate the authors for the excellent work they have presented [1]. It is pragmatic to recognize that a patient receiving high doses of vasopressors, who is tachycardic with low diastolic blood pressure, is critically ill and has a high mortality rate.

This study highlights two points that we must explore further. The first point is that a patient experiencing hypoperfusion events, defined by elevated lactate levels, with a diastolic pressure that might be considered adequate (e.g., 50 mmHg), moderate tachycardia of approximately 100 beats per minute, and low doses of norepinephrine equivalents at 0.05 mcg/kg/min, shows a 15% increase in mortality compared to the groups presented in the study (group 1 vs. group 3). Moreover, this last group has a 5% higher mortality rate compared to group 2, which received 35% higher doses of norepinephrine.

To find explanations for this increased mortality at low vasopressor doses, we must explore whether this group has a higher fluid balance than the other two groups due to the strategy of using more volume and fewer vasopressors. If this is true, the DAP-CVP gradient, which has already been associated in previous studies with greater hypoperfusion and renal failure [2]could explain why relatively adequate DAP levels end up being linked to a higher risk of mortality. If the hypothesis is suitable, this group would also have a higher number of cases with renal failure and the need for renal replacement therapy.

Another point we want to highlight is that age is an important factor with an odds ratio of 1.49, second only to the APACHE II score, which also includes this parameter. Thus, we present an adjustment to the proposed scale, incorporating the interaction of age, heart rate, and vasopressor dosage, which we have been working with.

Modified Eq. 1:

$$\:ME=\frac{DAP}{VO2max*NE}$$

Where VO2 max is defined as:

$$\:VO2=\frac{220-age}{actual\:HR}\:$$

In an exploratory analysis, this modification, which can still be relatively easily applied at the bedside, could increase the accuracy of the formula proposed by the authors by 25%.

We hope these observations will be useful for future studies.

## Data Availability

Not applicable.

## Abbreviations

APACHE II: Acute Physiology and Chronic Health Evaluation
CVP: Central Venous Pressure
DAP: Diastolic Arterial Blood Pressure
HR: Heart Rate
NE: Norepinephrine
VO2 max: Maximal Oxygen Consumption
